# Hexosamine Biosynthesis Is a Possible Mechanism Underlying Hypoxia’s Effects on Lipid Metabolism in Human Adipocytes

**DOI:** 10.1371/journal.pone.0071165

**Published:** 2013-08-14

**Authors:** Robert W. O’Rourke, Kevin A. Meyer, Garen Gaston, Ashley E. White, Carey N. Lumeng, Daniel L. Marks

**Affiliations:** 1 Department of Surgery, Oregon Health and Science University, Portland, Oregon, United States of America; 2 Department of Pediatrics, University of Michigan, Ann Arbor, Micigan, United States of America; 3 Department of Pediatrics, Oregon Health and Science University, Portland, Oregon, United States of America; University College Dublin, Ireland

## Abstract

**Introduction:**

Hypoxia regulates adipocyte metabolism. Hexosamine biosynthesis is implicated in murine 3T3L1 adipocyte differentiation and is a possible underlying mechanism for hypoxia’s effects on adipocyte metabolism.

**Methods:**

Lipid metabolism was studied in human visceral and subcutaneous adipocytes in *in vitro* hypoxic culture with adipophilic staining, glycerol release, and palmitate oxidation assays. Gene expression and hexosamine biosynthesis activation was studied with QRTPCR, immunofluorescence microscopy, and Western blotting.

**Results:**

Hypoxia inhibits lipogenesis and induces basal lipolysis in visceral and subcutaneous human adipocytes. Hypoxia induces fatty acid oxidation in visceral adipocytes but had no effect on fatty acid oxidation in subcutaneous adipocytes. Hypoxia inhibits hexosamine biosynthesis in adipocytes. Inhibition of hexosamine biosynthesis with azaserine attenuates lipogenesis and induces lipolysis in adipocytes in normoxic conditions, while promotion of hexosamine biosynthesis with glucosamine in hypoxic conditions slightly increases lipogenesis.

**Conclusions:**

Hypoxia’s net effect on human adipocyte lipid metabolism would be expected to impair adipocyte buffering capacity and contribute to systemic lipotoxicity. Our data suggest that hypoxia may mediate its effects on lipogenesis and lipolysis through inhibition of hexosamine biosynthesis. Hexosamine biosynthesis represents a target for manipulation of adipocyte metabolism.

## Introduction

Hypoxia is implicated as a cause of aberrant adipose tissue inflammation and metabolism in obesity [1]–[3]. Study of the effects of hypoxia therefore provides a model to identify mechanisms of adipocyte dysfunction. Hypoxia has diverse effects on cell metabolism but specific processes linking hypoxia to discrete metabolic functions in adipocytes are not well-defined. Hexosamine biosynthesis (HBS) has been implicated in adipocyte differentiation and systemic metabolic disease [4], [5]. Most data suggest that increased HBS is associated with increased insulin resistance in *in vitro* and *in vivo* systems [4], [6]–[9], although some studies demonstrate that HBS improves or has no effect on insulin resistance [10]–[12]. These data are primarily derived from murine systems and involve study of glucose homeostasis. The role of HBS in regulating lipid metabolism in adipocytes and its relationship to hypoxia are unknown, and data from human systems is sparse.

The goal of these experiments was to define the effects of hypoxia on human adipocyte lipid metabolism and identify underlying mechanisms. We hypothesized that hypoxia mediates its effects on adipocyte lipid metabolism through regulation of HBS. Our data demonstrate that hypoxia regulates lipid metabolism in human adipocytes and suggest a mechanistic link between HBS and hypoxia-induced alterations in lipogenesis and lipolysis.

## Methods

### Subjects, Ethics Statement

Obese subjects undergoing laparoscopic bariatric surgery were enrolled and written informed consent was obtained with OHSU Institutional Review Board approval consistent with applicable institutional and governmental regulations including the Declaration of Helsinki, as well as Title 45, US Code of Federal Regulations, Part 46, Protection of Human Subjects, revised Jan. 15, 2009, effective July 14, 2009. Visceral (greater omentum) and subcutaneous (abdominal wall) adipose tissues were harvested at the beginning of the operation and processed immediately. Tissue was collected from a total of 64 obese subjects undergoing bariatric surgery. Mean age was 47 years +/−13 S.D., mean BMI 49 kg/m^2^+/−10 S.D.; 70% of obese subjects were female. The prevalence of diabetes, hypertension, sleep apnea, hyperlipidemia, and gastroesopheal reflux disease were 44%, 45%, 42%, 47%, and 47% respectively. Medications included angiotensin converting enzyme inhibitor (9%), statin (23%), proton pump inhibitor (28%), NSAID (19%), beta blocker (20%), metformin (28%), and aspirin (28%).

### SVF Isolation, Adipocyte Differentiation, and Culture

All media and reagents were certified to have endotoxin levels less than 0.030 EU/ml. Vessels were dissected from adipose tissue, which was washed in PBS +2% BSA, minced, and further digested with Type II collagenase (175 units/ml in PBS +2% BSA, Life Technologies Inc., Carlsbad, CA, USA) for 60 minutes at 37°C with gentle agitation followed by centrifugation at 200 g for 10 minutes. The SVF cell pellet was retrieved and washed. SVF cells were plated at 400,000 cells per well in a 48-well plate and maintained in plating media (MEM Alpha Modification, 10% FBS, 1% penicillin/streptomycin) until confluence (2–3 days), then transferred to differentiation media (1∶1 DMEM:Ham’s F12) enriched with 100 nM dexamethasone, 500 nM human insulin, 200 pM triiodothyronine, and 540 µM IBMX for 14 days, after which differentiated adipocytes were used in experiments. Adipocytes were cultured in 21% O_2_ (normoxia) or 1% O_2_ (hypoxia) in a gas-impermeable chamber (Billups-Rothenberg, Inc., Del Mar, CA, USA) at 37°C. Azaserine (Santa Cruz Biotechnology Inc, Santa Cruz, CA, USA, Cat# SC-29063) and isoproterenol hydrochloride (Sigma-Aldrich Inc., St. Louis, MO, USA, Cat# I6504) were used at final concentrations of 10 µM and 3 µM respectively.

### Viability Assays

Propidium iodide nuclear staining (PI): adherent adipocytes were washed with PBS, and propidium iodide solution (2 ug/mL, Sigma Aldrich, Catalog #P4864) added. Cells were incubated for 5 minutes and absorbance read at 518–542 nm/573–608 nm (excitation/emission) on a Synergy 2 Multi-Mode Microplate Reader controlled by BioTek’s Gen5™ Reader Control and Data Analysis Software, with fluorescent reading capabilities. Relative fluorescent units (RFUs) were recorded. Hoechst dye staining: Hoechst dye 33342 (1 ug/mL, Life Technologies, Catalog #H3750) was added directly to media on adherent adipocytes and incubated for 30 minutes at 37°C. Cells were washed and absorbances read at 340–380 nm/440–480 nm (ex/em). XTT assay: XTT reagent (Biotium Inc, Catalog #30007) was added directly to culture media per manufacturer’s instructions and incubated for 2 hours at 37°C. Absorbance was read at 450 nm, with a background subtraction reading at 650 nm. Trypan blue exclusion: adherent adipocytes were washed with PBS and Trypan blue solution (1∶100 in PBS, Sigma Aldrich, Catalog #T8154) was added, followed by microscopy 5 minutes later. Percentages of total non-viable stained cells in viewing field were recorded.

### Lipid Assays

Adherent adipocytes in a 48-well plate were washed with PBS and 400 µl of PBS and 12 µl of AdipoRed reagent (Lonza Inc., Walkersville, MD, USA, Catalog# PT-7009) added. Plates were read on a Synergy 2 Multi-Mode Microplate Reader with fluorescent reading capabilities controlled by BioTek’s Gen5™ Reader Control and Data Analysis Software. AdipoRed uptake was measured at excitation and emission wavelengths of 485 nm and 590 nm respectively as a measure of lipogenesis.

Supernatants from adipocyte cultures were assayed for glycerol release as a measure of lipolysis using a spectrophotometry-based lipid metabolite assay kit (Sigma-Aldrich, Inc., St. Louis, MO, USA, Catalog# TR0100). Glycerol concentration (mg/ml) was determined based on a glycerol standard solution (Sigma-Aldrich Inc., St. Louis, MO, USA, Product #G7793). Per manufacturer’s instructions, absorbances were read at 540 nm on a Biomate 3 spectrophotometer (Fisher Scientific Inc., Newington, NH, USA).

Fatty acid oxidation (FAO) was measured by studying oxidation of ^3^H-palmitate based on a previously described assay [13]. Cells were incubated with or without stimulus in normoxia/hypoxia for 24 hours. The media was then removed and replaced with media enriched with non-tritiated palmitic acid (3 mM) and tritiated palmitic acid (9,10-^3^H(N), 10 µCi/mL, PerkinElmer, Inc., Waltham, MA, USA, Catalog #NET043001MC ) and incubated for 2 hours. Palmitic acid oxidation was assessed by measuring ^3^H_2_O release into the incubation medium. Media (200 µl) were extracted by addition of 1 mL of methanol/chloroform (1∶2) v/v) and 200 µL of 2 M KCL/HCL (1∶1, v/v), followed by centrifugation at 3,000 g for 15minutes. ^3^H_2_O release in the aqueous phase was measured by liquid scintillation counting (Beckman Coulter LS6500 Multi-Purpose Scintillation Counter).

### Western Blotting

Cell protein lysates in RIPA buffer (25–50 µg) were loaded on a 7.5% SDS-PAGE gel for electrophoresis then transferred to PVDF membrane. Membranes were blocked in TBST +5%BSA for one hour at 25°C, washed 3 times in TBST, then incubated in TBST +5%BSA for 12 hours at 4°C with primary antibodies specific for OGlNAc (Clone RL-2, Pierce Antibodies, product# MA1-072). Membranes were washed in TBST and incubated with IRDye800-conjugated goat anti-rabbit IgG (Rockland Immunochemicals, Inc., Gilbertsville, PA, USA) and Alexa Fluor 700-conjugated goat anti-mouse IgG (Invitrogen, Inc. Carlsbad, CA, USA) secondary antibodies in TBST/5%BSA for one hour at 25°C then washed. Parallel blots loaded with identical amounts of the same lysate preparations were probed with actin-specific antibody (R&D Systems, Minneapolis, MN, USA). Densitometry was performed using an Odyssey Infrared Imaging System and software (LI-COR Biosciences Inc., Lincoln, NE, USA). Densitometry data is normalized to actin levels.

### Immunofluoresence Microscopy

Adherent adipocytes in an IBIDI u-slide 8-well plate (ibidi GmbH, Martinsried, Germany) were fixed using 4% paraformeldahye. Cells were permeabilized with 0.25%Triton X-100, washed three times, blocked in a TBST+1% fish gelatin (Sigma-Aldrich, Inc., St. Louis, MO, USA, Catalog# G7765) +10% donkey serum (Sigma-Aldrich, Inc., St. Louis, MO, USA, Catalog# D9663) solution, and then incubated in TBST+1% fish gelatin for 12 hours at 4°C with primary antibodies specific for OGlNAc (Clone RL-2, Pierce Antibodies, product# MA1-072). Cells were washed three times, and incubated with Alexa Fluor 488 donkey anti-mouse IgG (Life Technologies, Inc., Carlsbad, CA, USA) secondary antibody in TBST +1% fish gelatin for one hour at 25°C, then washed three times. Nuclei were stained for 5 min using Hoeschst 33342 (Life Technologies, Inc., Carlsbad, CA, USA), washed, and visualized on a high resolution wide-field Core DV system (Applied Precision™) with an Olympus IX71 inverted microscope with a proprietary XYZ stage enclosed in a controlled environment chamber with differential interference contrast transmitted light and a solid state module for fluorescence, and a Nikon Coolsnap ES2 HQ camera (Nikon Inc., Tokyo, Japan). Z-stack images were transferred to Imaris, a multidimensional analysis program for analysis of fluorescence intensity data (Bitplane Inc., South Windsor, CT, USA). Surfaces were created based on A488 emission to track cells and extract fluorescent intensity. The intensity sum was divided by the number of cells in the viewing field (based on nuclei counted), and resulting average intensity per cell was reported.

### PCR

RNA was prepared from cells using an RNeasy lipid kit (Qiagen, Inc., Germantown, MD, USA) and treated with DNase. Equal amounts of input RNA were used for all reactions. RNA was reverse-transcribed using random hexamer primers. QRTPCR was performed using SYBR Green reagent and transcript-specific primers on an ABI7900 thermocycler (Applied Biosystems, Inc., Foster City, CA, USA). GAPDH and actin were used as endogenous controls and provided similar results in all cases. Fold changes relative to actin are reported. The 2^–ddCT^ relative quantification method was used to calculate fold difference in transcript levels between samples; efficiencies of amplification for all primer pairs were verified to be equivalent over a range of template concentrations.

### Primer Sequences

PPAR-γ: FOR: 5′AGCCTCATGAAGAGCCTTCCA3′REV: 5′TCCGGAAGAAACCCTTGCA3′

FAS:FOR: 5′CTGGCTCAGCACCTCTATCC3′REV: 5′CAGGTTGTCCCTGTGATCCT3′

ATGL:FOR: 5′GTGTCAGACGGCGAGAATG3′REV: 5′TGGAGGGAGGGAGGGATG3′

SREBP1c:FOR: 5′GGAGCCATGGATTGCACTTT3′REV: 5′TCAAATAGGCCAGGGAAGTCA3′

GFAT:FOR: 5′GGATATAGAATTTGATGTACACC3′REV: 5′GGGTGGCTATTGACAGGACTGG3′

OGT:FOR: 5′GCTGAGAGACACTGCATGCAGC3′REV: 5′CGACACTGGAAGTGTATAG3′

### Statistical Analysis

All statistical tests were two-tailed. All data were normally distributed. Paired t-tests were used to compare outcomes of all *in vitro* assays between normoxic and hypoxic arms, between media and azaserine or glucosamine arms, and between VAT and SAT adipocytes from the same subjects. Due to limited tissue and cell yields, different experiments utilized tissue from different groups of subjects. Delta CT values were compared for statistical analysis of QRTPCR data. Error bars in figures are standard error of the mean.

## Results

### Human Adipocyte Model

**Figure 1:**
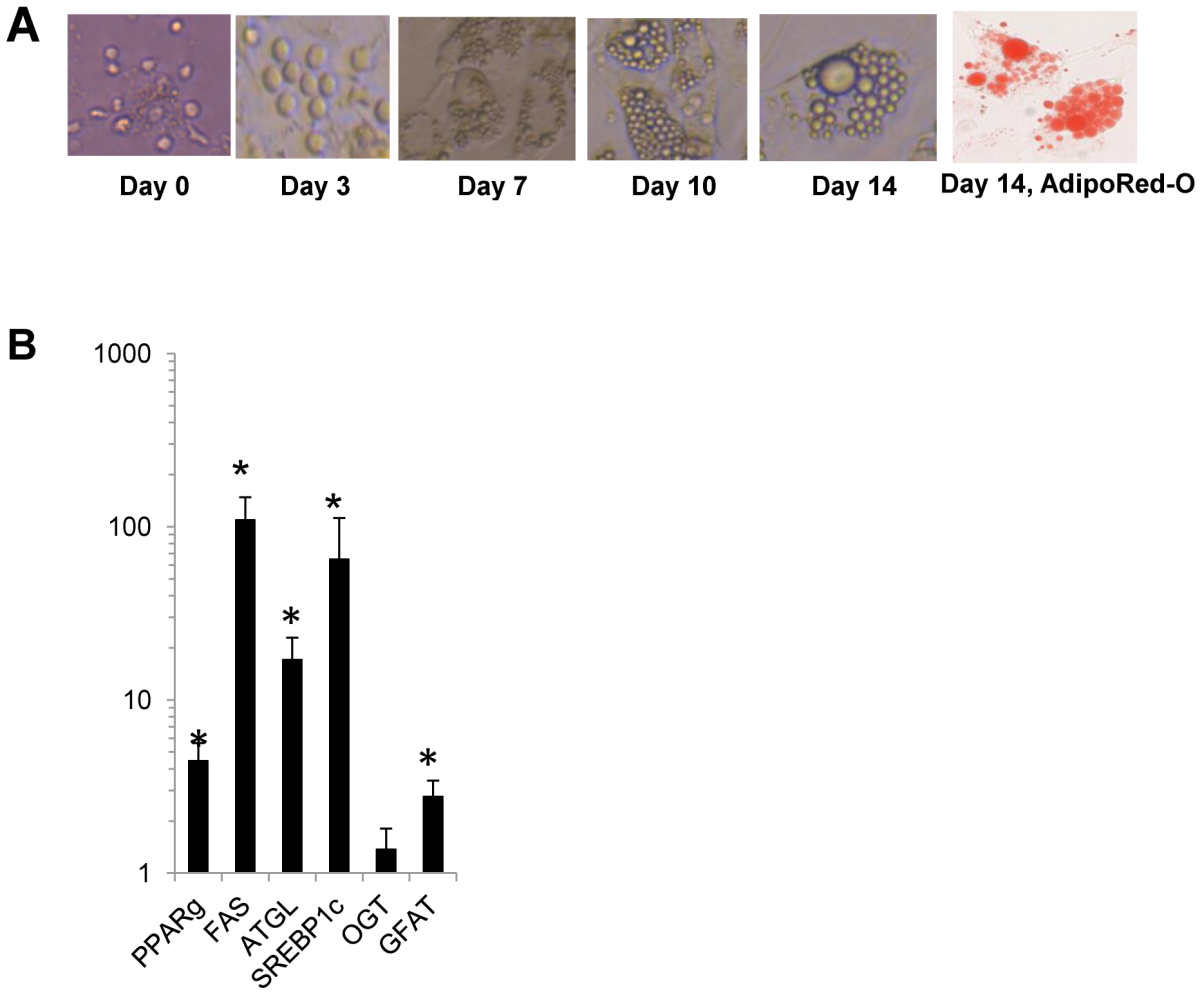
In vitro differentiated primary human adipocytes. A. Adipocyte differentiation: Representative light micrographs of human visceral adipocytes at various stages of differentiation with and without AdipoRed staining. Similar morphology and rates of differentiation were observed in adipocytes derived from subcutaneous adipose tissue. B. Adipogenic transcription: QRTPCR data comparing transcript levels in mature human visceral adipocytes relative to undifferentiated visceral SVF referent. Transcript levels of adipogenic genes PPAR-γ, fatty acid synthase (FAS), ATGL, and SREBP1c, as well as the rate-limiting HBS enzyme GFAT, markedly increased over the course of adipocyte differentiation. Ordinate is fold change in transcript level in mature adipocytes relative to undifferentiated SVF referent; asterisk: p<0.050, paired t-test, comparing transcript levels in mature adipocytes and SVF referent; data from adipocytes from n = 6 obese subjects.

Visceral and subcutaneous human adipocytes derived from SVF and differentiated in adipogenic medium over 14 days exhibit progressive accumulation of cytoplasmic lipid and increased transcript levels of genes associated with adipocyte metabolism, including PPAR-γ, fatty acid synthase (FAS), ATGL, and SREBP1c, as well as GFAT, the gene that encodes the rate-limiting enzyme involved in HBS. Transcript levels of OGT, a gene encoding a non-rate-limiting HBS enzyme, were not altered with adipocyte differentiation (**Figure 1**).

### HBS Increases during Human Adipocyte Differentiation

**Figure 2:**
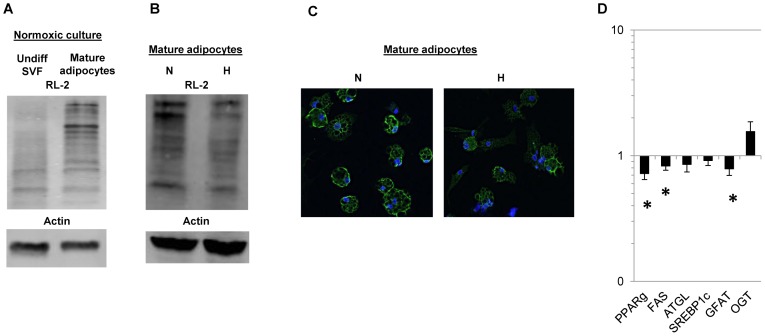
HBS is up-regulated during differentiation, and hypoxia inhibits HBS in human adipocytes. A. HBS increases during adipocyte differentiation: Representative Western blot comparing OGlcNAc levels measured by RL-2 antibody staining of protein lysates from undifferentiated visceral SVF and mature visceral adipocytes after 14 days of differentiation, with matched blot for same protein lysates probed with actin-specific antibody. Mean fold increase with differentiation determined by densitometry and normalized to actin levels = 2.00, SEM = 0.24, p = 0.025; paired t-test, n = 4 obese subjects. B. Hypoxia inhibits HBS in adipocytes: Representative Western blot comparing OGlcNAc levels measured by RL-2 antibody staining of protein lysates from mature visceral adipocytes cultured for 6 hrs in normoxic or hypoxic (N, H) conditions, with matched blot for same protein lysates probed with actin-specific antibody. Mean fold decrease with hypoxic culture relative to normoxic culture determined by densitometry and normalized to actin levels = 0.84, SEM = 0.04, p = 0.024, paired t-test, n = 8 obese subjects. C. Hypoxia inhibits HBS in adipocytes: Representative immunofluoresence microscopy comparing OGlcNAc levels measured by RL-2 antibody staining in mature visceral adipocytes cultured for 6 hrs in normoxic or hypoxic (N, H) conditions. RL-2-Green; nuclear stain: blue; mean fold decrease with hypoxic culture relative to normoxic culture referent determined by quantification of RL-2 immunfluoresence Z-stack microscopy signal normalized to cell number = 0.80, SEM = 0.04, p = 0.021, paired t-test,; n = 6 obese subjects**.**D. Hypoxia inhibits transcription of HBS and adipogenic genes in adipocytes: QRTPCR data comparing transcript levels in mature visceral adipocytes cultured in hypoxic conditions for 72 hours compared with matched visceral adipocytes cultured in normoxic conditions. Hypoxia decreased transcript levels of lipogenic genes PPAR-g and FAS, and the rate-limiting HBS enzyme GFAT. Hypoxia had no effect on transcript levels of ATGL, SREBP1c, or the non-rate-limiting HBS enzyme OGT. Ordinate: fold difference in transcript levels in cells cultured in N, H conditions; asterisk: p<0.050, paired t-test, comparing transcript levels in adipocytes cultured in N, H conditions; n = 7 obese subjects.

Given the observed increase in GFAT transcript levels with differentiation, we next studied HBS over the course of adipocyte differentiation. When compared to undifferentiated SVF, mature differentiated human visceral adipocytes demonstrated increased expression on Western blot analysis of adipocyte protein lysates with antibody specific for OGlcNAc, the glycosylation moiety that is covalently linked to multiple cellular proteins when HBS is activated, indicative of increased HBS over the course of adipocyte differentiation (**Figure 2A**).

### Hypoxia Inhibits HBS in Human Adipocytes

Given that hypoxia is implicated in adipose tissue dysfunction in obesity, we next studied the role of hypoxia in regulating HBS. Hypoxia inhibited HBS in mature human visceral adipocytes based on Western blot analysis of adipocyte protein lysates and immunofluorescence staining of mature adipocytes with antibody specific for OGlNAc (**Figure 2B, C**). Hypoxia also decreased transcript levels of the lipogenic genes PPAR-γ and FAS, as well as GFAT, the enzyme that mediates the rate-limiting step in HBS, while having no effect on transcript levels of SREBP1c, OGT, or ATGL, the rate limiting enzyme in lipolysis (**Figure 2D**).

### Hypoxia Inhibits and HBS Promotes Lipogenesis in Human Adipocytes

**Figure 3:**
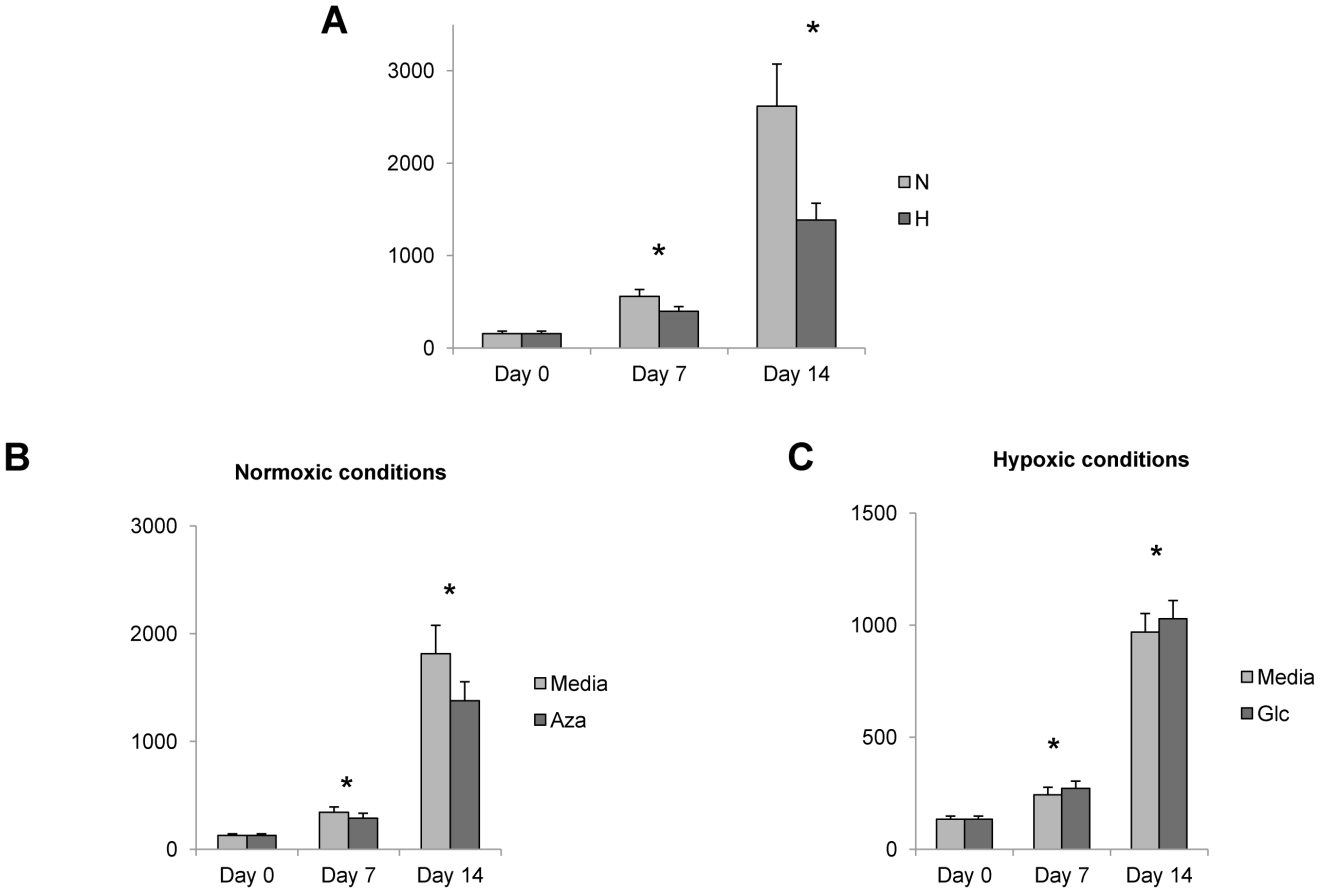
Hypoxia attenuates and HBS promotes adipocyte lipogenesis. A. Hypoxia inhibits adipocyte lipogenesis during differentiation: Lipogenesis measured by uptake of Adipo-Red reagent during visceral adipocyte differentiation in N, H conditions. Ordinate: Adipo-Red staining intensity determined by spectrometry normalized to cell number; asterisk: p<0.050, paired t-test, comparing N, H conditions; n = 6 obese subjects. Similar results were observed with subcutaneous adipocytes which demonstrated no quantitative difference in lipid accumulation at any time point c/w visceral adipocytes (data not shown). B. Azaserine inhibits lipogenesis in normoxic conditions: Visceral adipocytes were differentiated in normoxic conditions in the presence (Aza) or absence (media) of azaserine, a small molecule inhibitor of HBS. Azaserine inhibited lipogenesis over the course of adipocyte differentiation. Ordinate: AdipoRed staining intensity in arbitrary spectrophotometry units normalized to cell number; asterisk: p<0.050, paired t-test, comparing media, Aza arms; n = 7 obese subjects. C. Glucosamine slightly increases adipocyte lipogenesis in hypoxic conditions: Visceral adipocytes were differentiated in hypoxic conditions in the presence (Glc) or absence (media) of glucosamine, an HBS substrate and promoter of HBS. Glucosamine slightly increased adipocyte lipogenesis in hypoxic conditions. Ordinate: AdipoRed staining intensity in arbitrary spectrophotometry units normalized to cell number; asterisk: p<0.050, paired t-test, comparing media, Glc arms; n = 13 obese subjects.

Given the observed inhibition of the lipogenesis-related transcripts PPAR-γ and FAS in adipocytes in response to hypoxia, we next studied the effect of hypoxia on lipogenesis. Hypoxia inhibits lipogenesis over the course of differentiation in human visceral adipocytes based on AdipoRed staining (**Figure 3A**), with no effect on cell viability, based on viability assays using propidium iodide nuclear staining, Hoechst dye staining, XTT metabolism, and trypan blue exclusion (**data not shown**). Similar results with respect to lipid accumulation and viability were observed in subcutaneous adipocytes (**data not shown**).

Given the correlation of HBS with lipogenesis during differentiation, we next asked whether HBS was required for lipogenesis during adipocyte differentiation. Inhibition of HBS with the small molecule inhibitor azaserine attenuated lipogenesis independent of hypoxia (i.e. in normoxic conditions), consistent with a positive effect of HBS on adipocyte lipogenesis (**Figure 3B**). Similar results were observed in subcutaneous adipocytes (**data not shown**). Addition of glucosamine, a substrate for and promoter of HBS, slightly increased adipocyte lipogenesis in hypoxic conditions, (**Figure 3C**), also consistent with a lipogenic effect of HBS.

### Hypoxia Regulates Lipolysis and FAO Human Adipocytes, and HBS Inhibition Induces Basal Lipolysis

**Figure 4:**
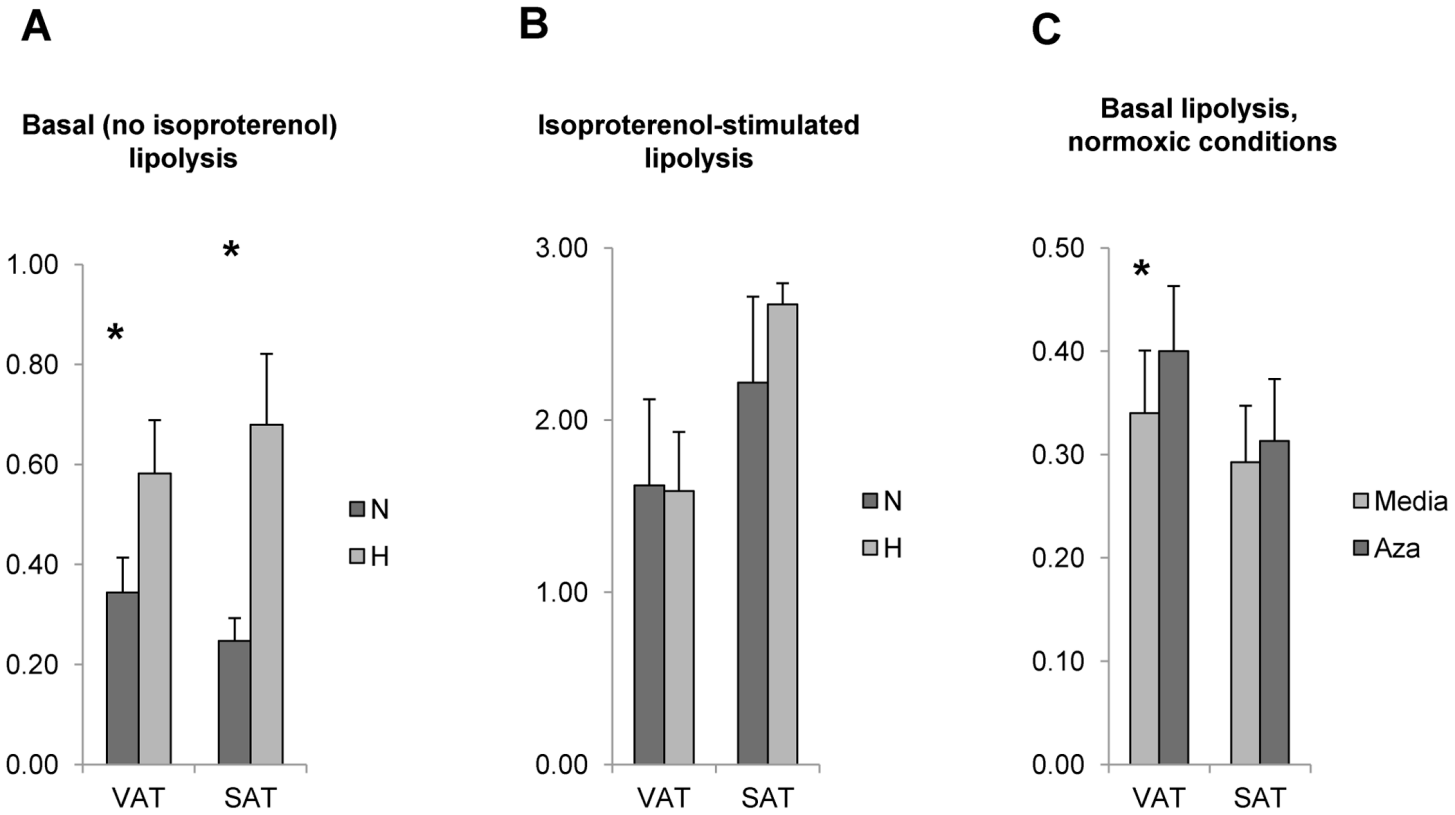
Hypoxia induces basal lipolysis in human adipocytes independent of HBS. A. Hypoxia induces basal lipolysis in human adipocytes: Mature visceral (VAT) or subcutaneous (SAT) adipocytes were cultured in N, H conditions for 24 hrs, then glycerol release measured in supernatants with a spectrophotometric assay kit. Hypoxia induced basal lipolysis in visceral and subcutaneous adipocytes. Ordinate: glycerol concentration (mg/ml); asterisk: p<0.050, paired t-test, comparing N, H conditions; n = 10 obese subjects. B. Hypoxia does not regulate b-adrenergic stimulated lipolysis in VAT adipocytes: Mature VAT adipocytes were cultured in N, H conditions for 24 hrs with 3 mM isoproterenol then glycerol release measured in supernatants with a spectrophotometric assay kit. Hypoxia had no effect on isoproterenol-stimulated lipolysis in adipocytes. Ordinate: glycerol concentration (mg/ml); asterisk: p<0.050, paired t-test, comparing N, H conditions; n = 10 obese subjects. C. HBS does not regulate basal lipolysis in human adipocytes: Mature adipocytes were cultured in normoxic conditions for 24 hrs in the presence or absence of the HBS-inhibitor azaserine and supernatants studied for glycerol release. Ordinate: glycerol concentration (mg/ml); no statistically significant difference between media, azaserine arms for VAT or SAT adipocytes; n = 10 obese subjects.

**Figure 5:**
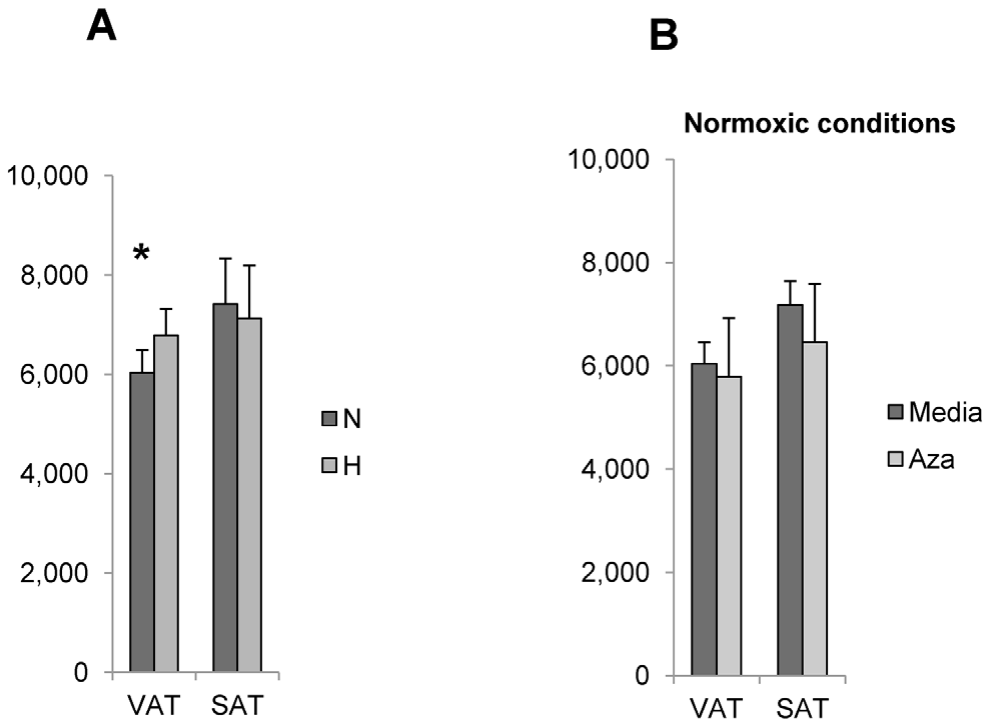
HBS regulates FAO in visceral but subcutaneous adipocytes independent of HBS. A. Hypoxia induces FAO in VAT but not SAT adipocytes: Mature visceral (VAT) or subcutaneous (SAT) adipocytes were cultured in N, H conditions for 24 hrs, then pulsed with ^3^H-palmitate and release of ^3^H2O into supernatants studied with scintillation counting. Ordinate: counts per minute normalized to cell number; p<0.050, paired t-test, comparing N, H conditions; n = 11 obese subjects. B. HBS does not regulate fatty acid oxidation in human adipocytes: Mature adipocytes were cultured in normoxic conditions for 24 hrs in the presence (aza) or absence (media) of azaserine, pulsed with ^3^H-palmitate, and release of ^3^H2O into supernatants studied with scintillation counting. Ordinate: counts per minute normalized to cell number; no statistically significant difference between media, azaserine arms for VAT or SAT adipocytes; n = 11 obese subjects.

We next studied the role of hypoxia and HBS in regulating lipolysis. Hypoxia induced basal but not isoproteronol-stimulated lipolysis in visceral and subcutaneous adipocytes. Inhibition of HBS with azaserine increased basal lipolysis in normoxic conditions in adipocytes from visceral adipose tissue but not in adipocytes from subcutaneous adipose tissue (**Figure 4**). Hypoxia induced a modest increase in FAO in visceral adipocytes but had no effect on FAO in subcutaneous adipocytes. Inhibition of HBS with azaserine had no effect on FAO in normoxic conditions (**Figure 5**).

## Discussion

### Hypoxia and Adipocyte Lipid Metabolism

Hypoxia has predominantly detrimental effects on adipocyte metabolism. Hypoxia induces insulin resistance in murine 3T3L1 adipocytes and adipose tissue hypoxia is associated with obesity and systemic insulin resistance in *in vivo* murine systems [3], [14], [15]. The effects of hypoxia on adipocyte lipid metabolism are not well-established. We demonstrate that hypoxia inhibits lipogenesis and induces basal lipolysis in human adipocytes, while having depot-specific effects on FAO. We also implicate HBS as a mechanism underlying hypoxia’s effects on adipocyte lipid metabolism.

Our findings with respect to hypoxia’s effects on lipogenesis and adipogenic transcription in human adipocytes are consistent with data from 3T3L1 cells and a single published study of human subcutaneous adipocytes that demonstrate hypoxia-induced suppression of lipogenesis along with suppression of adipogenic transcription factors PPAR-γ and C/EBPβ [16]–[20]. Sparse previously published data study the effects of hypoxia on adipocyte lipolysis, which also support our findings: hypoxia induces lipolysis in 3T3L1 adipocytes [15], [21], while studies in humans confirm a correlation between adipose tissue hypoxia and systemic resistance to insulin’s anti-lipolytic effects [22]. We are not aware of prior data studying FAO in response to hypoxia in adipocytes, but our data supports a modest depot-specific positive effect of hypoxia on FAO in human adipocytes.

**Figure 6:**
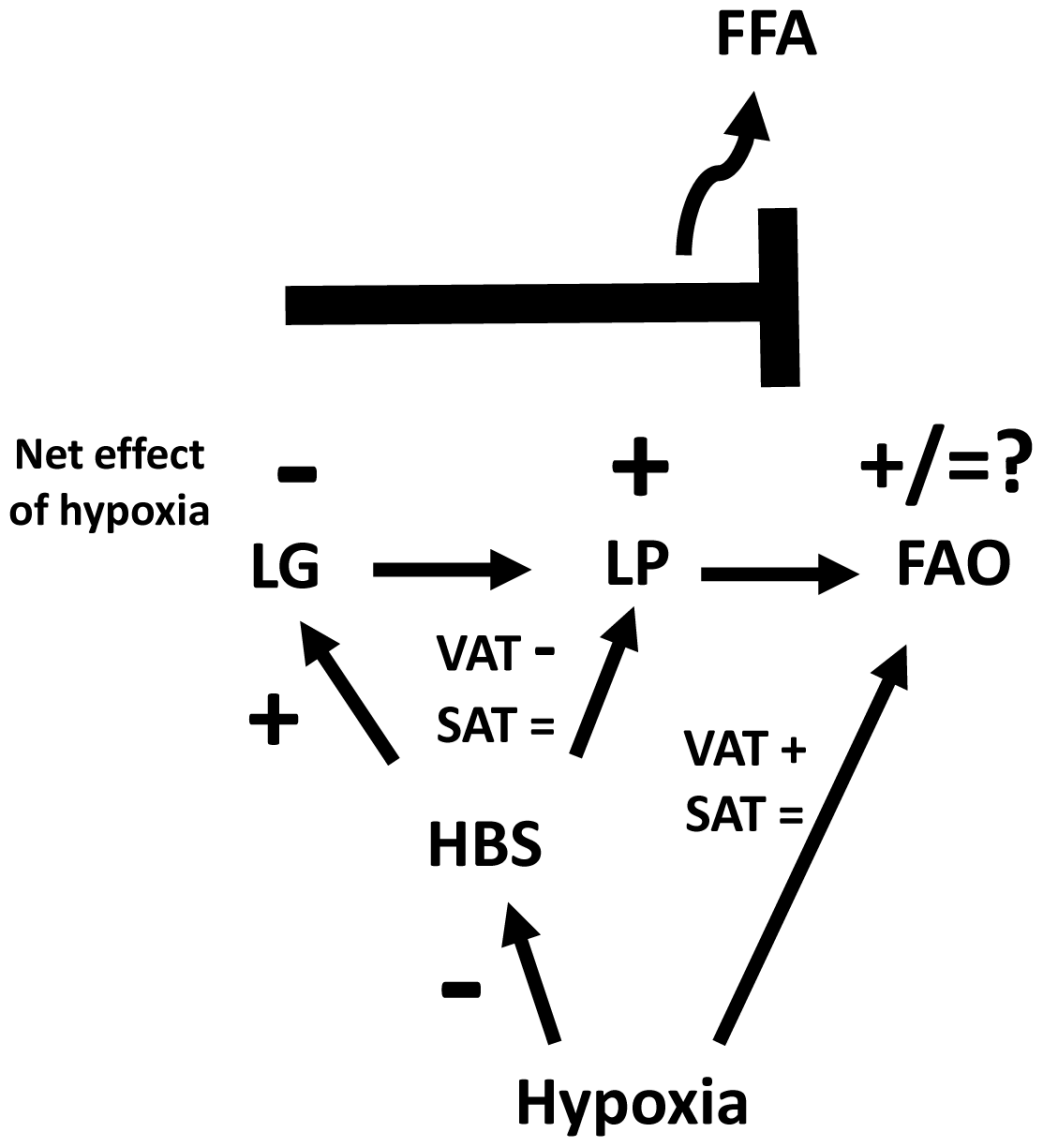
A model for hypoxia/HBS-mediated adipocyte overflow. HBS induces lipogenesis (LG) and inhibits lipolysis (LP) promotes FAO in VAT but not SAT adipocytes. Hypoxia also inhibits HBS. HBS in turn promotes LG and inhibitis LP in VAT but not SAT adipocytes. The net effect of hypoxia is to inhibit LG and induce LP. This shifts lipid metabolism towards LP, inhibiting adipocyte lipid storage and buffering capacity, increasing free fatty acid (FFA) release, and thus promoting systemic lipotoxicity. Depot-specific differences in the magnitude and direction of these responses add complexity.

Complex processes such as hypoxic responses likely generate both adaptive and maladaptive effects, and caution must be exercised in extrapolating *in vivo* systemic effects from *in vitro* study of isolated cells. Nonetheless, the net result of hypoxia-induced inhibition of lipogenesis and induction of lipolysis would be impaired adipocyte lipid buffering capacity and increased FFA production. These local adipocyte effects would be expected to translate *in vivo* into overflow of free fatty acids into the systemic circulation and promote lipotoxicity (**Figure 6**). In support of this concept, acute *in vivo* hypoxic challenge in mice induces adipose tissue lipolysis and results in systemic hyperlipidemia [23]. Furthermore, femoral artery clamping rats as an *in vivo* model of tissue hypoxia is associated with an increase in serum FFA [21]. Finally, obesity is associated with increased basal (but not β-adrenergic-stimulated) lipolysis in human adipocytes [24], [25], consistent with our *in vitro* observations in response to hypoxia, suggesting that hypoxia may play a role in obesity’s effects on adipocyte lipolysis *in vivo*.

In conflict with our hypothesis however, many murine models of enhanced lipolysis manifest improved metabolism [26]–[29], although exceptions exist [30]. Importantly, depending on the genetic manipulation, most transgenic *in vivo* models of reduced lipolysis are associated with other alterations in lipid metabolism, including increased FAO [26]. Our *in vitro* data demonstrate that hypoxia induces a modest increase FAO in visceral adipocytes but has no effect on FAO in subcutaneous adipocytes. While increased FAO is not consistent with our hypothesis of hypoxia-induced increased lipid overflow, it is important to note that relative to its effects on lipolysis, hypoxia exerted modest effects on FAO in visceral adipocytes and had no effect on FAO in subcutaneous adipocytes. Rather than any single aspect of lipid metabolism, it is net cellular lipid flux and the relative balance of lipogenesis, lipolysis, and FAO that determine adipocyte lipid output. Furthermore, systemic *in vivo* metabolic effects are dictated by the sum of the contributions of all adipose tissue depots, which manifest quantitative and qualitative differences in metabolism to a range of stimuli, including hypoxia, as our data demonstrate. The significance of these depot-specific differences in hypoxic responses at the systemic level is unclear, but in contrast to lipolysis and lipogenesis which were similarly regulated by hypoxia in adipocytes from both depots, the magnitude of the FAO response to hypoxia was modest in visceral adipocytes and absent in subcutaneous adipocytes, suggesting that with respect to total body adipose tissue stores, hypoxia instead has a more important role in regulating lipolysis and lipogenesis. A proportionally greater hypoxia-induced inhibition of lipogenesis and induction of lipolysis compared to induction of FAO, as observed in our data, would be consistent with increased lipid overflow from adipose tissue and shunting of lipid to other tissues.

### HBS as an Underlying Mechanism of Hypoxia’s Effects on Adipocyte Lipid Metabolism

We demonstrate that hypoxia inhibits HBS, and that inhibition of HBS mimics hypoxia’s effects on adipocyte lipogenesis and basal lipolysis in normoxic conditions, inhibiting lipogenesis and inducing lipolysis. Furthermore, promotion of HBS with glucosamine slightly increased adipocyte lipogenesis in hypoxic conditions. These observations are consistent with prior data which demonstrate that inhibition of HBS impairs lipogenesis in murine adipocytes [5], [30], [31]. In contrast to our data, however, prior data demonstrate a positive effect of HBS on FAO in murine adipocytes [12], [30]. This discrepancy may reflect differences in human and murine systems. As far as we know, no data studies the role of HBS in regulating lipolysis in adipocytes. The effects of HBS inhibition applied to both visceral and subcutaneous adipocytes in the case of lipogenesis, but only to visceral adipocytes in the case of lipolysis, suggesting qualitative depot-specific differences in the role of HBS in regulating lipolysis, similar to the observed depot-specific differences in hypoxia’s regulation of FAO. These observations also suggest that unlike visceral adipocytes, subcutaneous adipocytes regulate hypoxia-induced lipolysis via mechanisms other than HBS. Taken together, these findings suggest that hypoxia mediates some of its effects on adipocyte lipid metabolism via inhibition of HBS.

Data is conflicting regarding whether HBS is metabolically detrimental or beneficial at the systemic level [4]–[12], and like hypoxia, HBS likely has both adaptive and maladaptive effects. Our data suggest that HBS promotes adipocyte lipid storage and inhibits free fatty acid release, consistent with a beneficial role for HBS with respect to adipocyte lipid metabolism via promotion of adipocyte buffering capacity. Furthermore, our data also suggest that hypoxia mediates its detrimental effects on adipocyte lipid metabolism in part through inhibition of HBS.

### Other Considerations

Hypoxia’s effects on adipocyte metabolism may be due to effects on cell viability. Our data demonstrate no effect of 1% O_2_ culture on human adipocyte viability over the course of differentiation, suggesting that at least in this *in vitro* culture system, hypoxia’s effects on adipocyte metabolism do not involve viability. These observations are consistent with data from others that show no effect or increased viability in response to hypoxia in human adipocyte precursors [30], [32], suggesting that adipocytes are to some extent adapted to survival at lower oxygen levels.

*In vivo* tissue O_2_ concentrations, while not well-established, are lower than standard tissue culture concentrations of 21%, and 1% O2 *in vitro* hypoxic culture only approximates pathogenic *in vivo* conditions. Twenty-one percent and one percent are commonly employed to approximate “normoxia” and “hypoxia” *in vitro*, however, and 1% O_2_ induces marked inflammatory and metabolic responses [1]–[3], [33]–[38]. Thus, while not an exact simulation of *in vivo* hypoxia, *in vitro* hypoxic culture nonetheless provides a useful model system. Similarly, the *in vitro* human adipocyte culture system provides only an approximation of *in vivo* phenotype and function. Adipocytes differentiated in this manner are nonetheless a well-accepted model and retain depot and host-specific characteristics in culture [39].

Some observed responses, including the fold decrease of HBS in response to hypoxia, the increase in lipolysis in response to azaserine, and the depot-specific regulation of FAO by hypoxia, were modest in magnitude, but nonetheless reproducible and statistically significant. These low magnitude effects may be explained in part by the intrinsic heterogeneity of human adipocyte cultures, which range from 60–90% differentiation efficiency. In addition, variability between patient samples contributed to the low magnitudes of some signals, but with paired analysis, these differences were nonetheless statistically significant. Future experiments studying adipocytes derived from dedicated purified precursor subpopulations from subjects matched for clinical variables may provide more homogenous populations and increase assay signal. Finally and importantly, the effect of hypoxia on HBS was modest, mediating only a 0.8-fold decrease in HBS. Nonetheless, this effect was reproducible, statistically significant, and identical with two different assays (Western blotting and immunofluorescence microscopy). Furthermore, the effects of azaserine and glucosamine on adipocyte lipid metabolism, while statistically significant, were of similarly low magnitudes. These low magnitude effects suggest that HBS is likely one of many factors that mediates hypoxia’s effects on adipocyte lipid metabolism. Off-target effects of azaserine and glucosamine may further contribute to these modest effects. Future research will determine the importance of HBS in mediating hypoxia’s effects on adipocyte metabolism.

Limitations in human subjects, tissue availability, and cell yield precluded analysis of all aspects of lipid metabolism including the effects of known mediators of lipolysis and FAO in the context of hypoxia, such as insulin, adiponectin, and other stimuli, as well as rigorous correlation of *in vitro* results with clinical characteristics such as the presence of metabolic disease. Future research will be dedicated to studying these details of lipid metabolism.

### Conclusions

We demonstrate that hypoxia shifts adipocyte lipid metabolism towards a pro-lipolytic, anti-lipogenic phenotype, an effect that would be expected to impair lipid buffering capacity and predispose to systemic lipotoxicity. Furthermore, we demonstrate that down-regulation of HBS is a potential underlying mechanism of hypoxia-mediated alterations in human adipocyte lipid metabolism. These observations identify HBS-related mediators and other hypoxia-inducible molecules as targets to regulate adipocyte metabolism.
